# Differential and shared effects of psychological skills training and mindfulness training on performance-relevant psychological factors in sport: a randomized controlled trial

**DOI:** 10.1186/s40359-020-00449-7

**Published:** 2020-08-06

**Authors:** Philipp Röthlin, Stephan Horvath, Severin Trösch, Martin grosse Holtforth, Daniel Birrer

**Affiliations:** 1 Swiss Federal Institute of Sport, Alpenstrasse 18, CH-2532 Magglingen, Switzerland; 2 Institute for Psychology, University of Bern, Bern, Switzerland; 3 Competence Center Psychosomatic, Inselspital, Bern, Switzerland

**Keywords:** Sport psychology, Intervention, Randomized controlled trial, Psychological skills training, Mindfulness, Competitive sport, Athletic performance, Bayesian analysis

## Abstract

**Background:**

Mental training intends to support athletes in mastering challenges in sport. The aim of our study was to investigate the differential and shared effects of psychological skills training and mindfulness training on psychological variables relevant to athletic performance (e.g., handling emotions or attention control). We assumed that each approach has its own strengths (e.g., mindfulness has a differential effect on the acceptance of emotions), but for some goals (e.g., attention control), both training forms are expected to be equally successful (i.e., shared effects).

**Methods:**

A total of 95 athletes (*M*_age_ = 24.43, *SD*_age_ = 5.15; 49% female) were randomly assigned into three groups: psychological skills training intervention (PST), mindfulness training intervention (MT), and wait-list control group (WL). Participants completed a questionnaire battery before and after the training (pretest and posttest). We assessed mindfulness, use of mental strategies, handling of emotions, attention in training and competition, as well as the dealing with failure. The two intervention programs each consisted of four 90-min group workshops conducted over a period of 4 weeks.

**Results:**

Both interventions passed the manipulation check, that is, PST led to more mental strategies being used (probabilities > 95%), and MT led to an increase in two of three aspects of mindfulness (probabilities > 98%) when compared to WL. Compared to WL, both interventions equally improved in the ability to not let emotions interfere with performance (probabilities > 99%) and in controlling attention in training and competition (probabilities > 89%). To a lesser extend, both interventions showed shared improvements in dealing with failure indicated by more action orientation (probabilities > 82%). We found a differential effect of MT on decreased experiential avoidance: MT decreased compared to WL and PST (probabilities > 92%), whereas PST did not differ from WL.

**Conclusion:**

We conclude that both forms of mental training lead to improvements in performance-relevant psychological factors, especially concerning the handling of emotions and attention control. The results of our study suggest that different paths may lead to the desired outcomes, and accordingly, both forms of mental training seem justified.

**Trial registration number:**

ISRCTN11147748, date of registration: July 11, 2016.

## Background

Mental training in sport aims to help athletes better deal with the challenges of competition and training [39]. Among such challenges are, for example, mastering a crucial game situation at the most important tournament of the year or achieving a high quality of training in repetitive and possibly boring training sequences. Two well-known forms of mental training are psychological skills training and mindfulness training. The two forms of mental training each have a different theoretical background. Psychological skills training relies on methods of the first and second wave of cognitive behavioral interventions whereas mindfulness training is a central method of the third wave [6]. Interventions of the third wave differ from those of the first and second waves primarily in that they focus on changing the relationship to one’s own experience and less, as in the first and second waves, on the experience itself [20]. There are also similarities and overlaps between the two forms of mental training, for example, both lead athletes to engage with and reflect on their own experience. Typical techniques of the first and second wave are, for example, relaxation, reformulating negative self-talk into positive self-talk, or reframing [19, 39]. A central component of all interventions of the third wave is mindfulness, which describes the ability to face the current experience from a certain distance in an open and accepting way without trying to change it [25]. In the present study, we examined whether both approaches are applicable and whether each approach is associated with differential outcomes.

The goal of our study was to investigate the effects of a mindfulness training intervention (MT) and a psychological-skills training intervention (PST) on psychological variables that are relevant to athletic performance. A central point of our study was that we were not interested in determining which of the two forms of mental training was generally “better”. Instead, we hypothesized that different forms of mental training have different effects. We assumed that each approach has its unique strength (i.e., differential effects), but for some goals, both can be applied successfully (i.e., shared effects). We were thus interested in a wider range of effects of the two mental trainings and therefore included various psychological variables relevant to athletic performance (e.g., dealing with emotions or attention) to show differential *and* shared effects. Two reviews illustrate why these psychological variables are performance relevant and how psychological skills training and mindfulness training might improve them: Birrer and Morgan [5] presented a framework on how psychological techniques may affect psychological skills, which are necessary to satisfy sport-specific requirement. This framework included typical psychological skills training techniques, namely self-talk, imagery, goal-setting, and activation/relaxation, but also mindfulness. However, in contrast to the psychological skills training components, mindfulness rather represents a meta-technique, which influences athletic performance through different mechanisms. Accordingly, Birrer et al. [7] extended the framework by proposing nine potential impact mechanisms (e.g., experiential acceptance or less rumination), which were especially proposed as impact mechanisms for mindfulness training. We have already published the theoretical background and the design of our study in a study protocol [31].^1^ In this paper, we present the results.

While there are reviews and meta-analyses that have examined the effectiveness of either psychological skills training or mindfulness in sport settings (e.g., [11, 28]; for an overview see [31]), very few studies have compared the effectiveness of psychological skills training and mindfulness. We would like to mention two of them [18, 24]. Both studies, compared a mindfulness- and acceptance-based intervention with a psychological skills training intervention. Gross et al. [18] investigated a sample of 22 female NCAA Division III basketball players and found, amongst other things, that the mindfulness group reported better emotion-regulation skills when compared with the psychological skills training group. No group-by-time interactions were found on experiential avoidance and mindfulness, which may be due to the small sample size and the associated low statistical power of the study. Josefsson et al. [24] investigated a sample of 69 athletes from five different team and individual sports. They showed that the mindfulness group increased dispositional mindfulness and emotion regulation compared to the psychological skills training group and that these increases, in turn, led to improved self-rated athletic performance. Our study ties in with the studies by Gross et al. [18] and Josefsson et al. [24] but goes beyond it in two important ways: First, we included a nontreatment control group, which allowed us to evaluate if outcomes were due to treatment effects or the passage of time. Second, we did a manipulation check not only for the MT but also for PST.

We conducted a randomized controlled trial (RCT) with two intervention groups (MT and PST) and a wait-list control group (WL) in a sample of competitive athletes. Our research questions can be divided into four main parts: (1) manipulation check of the interventions, and outcome variables that focus on (2) handling of emotions, (3) attention and cognition, and (4) how athletes cope with failure. Concerning the manipulation check, we hypothesized that participation in intervention programs (MT or PST) would lead to more mindfulness and use of mental techniques, respectively.

We expected participants of MT to report being better in dealing with emotions in training and competition, an important requirement for good athletic performance. Since psychological skill training techniques are also used to deal with emotions (e.g., through positive thoughts or relaxation techniques), and not letting emotions prevent athletes from performing, we assumed that PST would have a similar positive effect. Thus, we hypothesized that the two interventions would not differ concerning the ability to prevent emotions from interfering with performance, but would be better in this respect than the WL. On the other hand, we assumed that PST and MI have different effects on certain ways of dealing with emotions, namely when it comes to not avoiding them. Not avoiding experiences has been proposed to be an impact mechanisms for mindfulness training [7]. Accordingly, we hypothesized that, compared with participants in PST or WL, participants in MT would report decreased experiential avoidance and an increased ability to accept emotions, as these components are central aspects of mindfulness based interventions [20]. The willingness to stay in contact with unpleasant experiences is closely connected with acceptance of emotions, both making maladaptive ways of emotion regulation less likely (e.g., through avoidance, [17]), so that more attentional resources are available for the current athletic task at hand [15].

Concerning attention and cognition, we hypothesized that participants in both intervention groups would improve their ability to control attention in training and competition compared with the WL. We also assumed that improved attention would be associated with a reduction of disturbing thoughts and negative cognitions in both groups (i.e., cognitive interference). Psychological skills, such as goal setting and self-talk, may serve as strategies to focus attention [38, 40], and mindfulness has proven to generally improve attention [12]. Improvements in attention are hypothesized to be an impact mechanism for psychological skills training and mindfulness by helping athletes to concentrate on the task at hand in the presence of potential internal and external distractors, and over a long period of time [5, 7]. While we expected improvements in attention control and cognitive interference in both interventions, we assumed that decentering would only improve in MT. Decentering, which is sometimes referred to as metacognitive awareness or defusion, is a central aspect of mindfulness-based interventions and consists of the ability to observe one’s thoughts and emotions from the distance and view them as passing mental events rather than identifying with them [21]. This clarity about once internal events possibly helps athletes because it could allow them to be more flexible in dealing with, for example, unhelpful thoughts and respond to them less automatically and is hypothesized to be an impact mechanism of mindfulness training [7]. In this context “flexible” means being able to decide when to pay attention to a thought (namely, if the thought is helpful) and when not to pay attention (namely, if the thought is not helpful).

As far as dealing with mistakes is concerned, we looked at action vs. state orientation after failures in sport. Action orientation characterizes a quick refocusing after failure, and sometimes mistakes are even motivating. In contrast, state orientation describes a longer time to dwell when an error occurs (e.g., by ruminating about what happened, [1]). In most cases, this kind of sport specific rumination after failure [26] is a disadvantage for sport performance [2]. Athletes with an action orientation can handle failures in high demanding situations more efficiently and draw the attention to forthcoming challenges. Therefore, in competitions, action orientation is often considered the better response than state orientation. We assumed that both interventions would be associated with better action orientation vs. state orientation than the WL. Less rumination is thought to be a another impact mechanism in mindfulness training [7]. Accordingly, mindfulness can lead to an improved action orientation and reduced state orientation by helping athletes to a) quickly realize maladaptive processes such as ruminating, b) be able to let go of these processes, and c) refocus their attention on more adaptive cues [22]. Psychological skills training might increase action orientation through the help of process goals, for example, an athlete could set a process goal to focus quickly on the next situation after a mistake [40]. Therefore, PST and MT should not differ with respect to action vs. state orientation, but be better than the WL.

## Methods

### Study design

We used a four-week RCT design to investigate the effect of two types of mental training on various outcome variables that are relevant to athletic performance. We used a 3 group (MT, PST, and WL) × 2 time points (pretest, posttest) design. Pre-post-changes in mindfulness and use of psychological techniques served as a manipulation check for our interventions. Outcome variables were the handling of emotions (i.e., experiential avoidance, acceptance of emotions, and the ability to not let emotions interfere with performance in training and competition); attention and cognition (i.e., attentional control in training and competition, cognitive interference, and decentering); and dealing with failures in competition (i.e., action orientation and state orientation).

### Participants and allocation

**Fig. 1:**
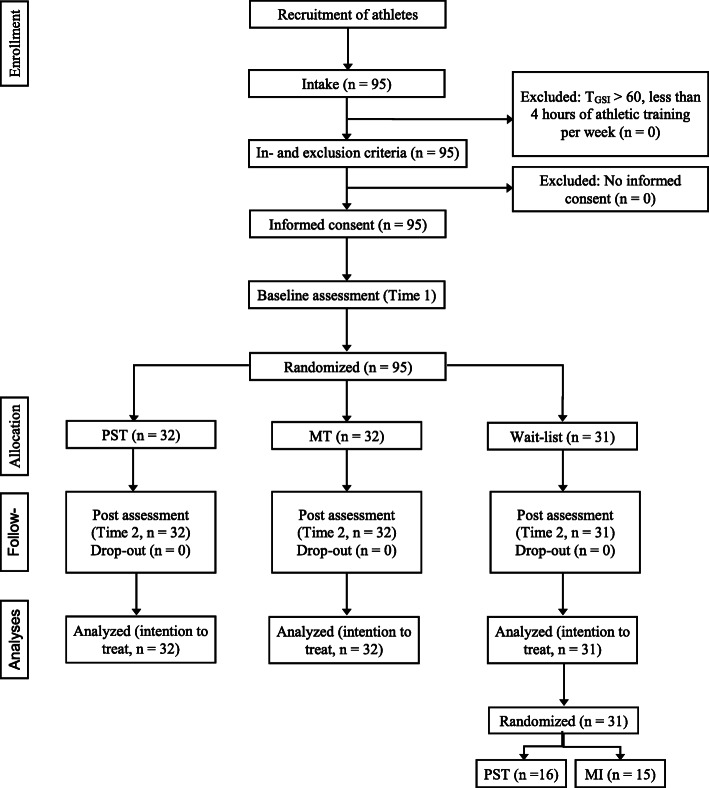
Participant recruitment and flow through the study

We recruited 95 athletes from four sports through contacts with their respective sport federations (tennis, curling, floorball, and badminton). The federations were asked to forward the call for study participation to athletes who met eligibility criteria, namely, a minimum age of 18 years and a minimum of four training hours per week. Athletes who wanted to participate were instructed to contact the authors by e-mail, who then sent the participants a link to an online survey (pretest of all study variables). No participant had to be excluded from the study due to a significant level of psychopathology; all participants scored below the Brief Symptom Inventory (BSI) threshold of 60 ([36], range 18–42, M = 26.12, SD = 5.51). After the pretest, participants were stratified by gender and sport and randomly allocated to either the MT, PST, or WL groups. The sample included six curling teams (3 male/3 female) and seven floorball teams (3 male/4 female). Considering ecological validity and feasibility in a competitive sport context, we allocated athletes who were members of the same curling or floorball team to the same group. After grouping, the participants were informed about their group membership. A CONSORT study flow diagram is shown in Fig. 1. All participants provided written informed consent prior to participation in the study. The study was approved by the Institutional Review Board of the Swiss Federal Institute of Sport Magglingen.

All participants who completed the pretest also completed the post assessment (i.e., there were no dropouts). Table 1 gives an overview of the study sample. There were no differences among the groups in age, gender, or sport. However, the PST group reported fewer training hours per week. No differences among groups were observed in any of the outcome variables on the pretest (*F*s < 2.44, *p*s > .09, all ${\upeta}_{\mathrm{p}}^2$ < .05; all Bayes Factors (BF) < 3, [23])

**Table 1:** Description of the study sample

Variable	Total (*n* = 95)	MT group (*n* = 32)	PST group (*n* = 32)	WL group (*n* = 31)	Group differences
Age (*M*/*SD*)	24.43 (5.15)	23.84 (5.51)	24.47 (5.27)	25.00 (4.74)	*F* = 0.42, *p* = .66
Sex	48 m/47f	17 m/15f	17 m/15f	14 m/17f	*Χ*^2^ = 0.52, *p* = .67
Sport	24 Curling	8 C (4 m/4f)	8 C (4 m/4f)	8 C (4 m/4f)	*Χ*^2^ = 0.19, *p* = .91
	48 Floorball	17 F (8 m/9f)	15 F (6 m/9f)	16 F (6 m/10f)	
	21 Tennis	7 T (5 m/2f)	8 T (7 m/1f)	6 T (4 m/2f)	
	2 Badminton	0 B	1 B (0 m/1f)	1 B (0 m/1f)	
Training hours per week	7.94 (3.25)	9.00 (3.28)	6.94 (3.22)	7.87 (2.98)	*F* = 3.42, *p* = .04

*Note.* m = male, f = female, C = curling, F = floorball, T = tennis, B = badminton

### Intervention and procedure

The MT and PST intervention programs consisted of four 90-min group workshops held over a period of 4 weeks (one workshop per week). For a detailed description of the contents of the workshops see Röthlin and Birrer [30]. The goal of the MT was to teach participants mindfulness. The goal of the PST program was to teach participants four mental techniques (i.e., arousal regulation, imagery, self-talk, and goal setting). Both interventions aimed to teach athletes in a way that enabled them to apply mindfulness or mental techniques during their training and in competitions. The workshops consisted of psychoeducation, hands-on exercises, self-paced worksheets, and opportunities to share thoughts and ask questions. Wherever possible, we used pictures, videos, and graphics to illustrate the learning content. Between the workshops, all participants completed short homework assignments. In addition, the participants were given a formal exercise to practice at home using an audio file. The formal exercises lasted around 10 to 12 min and were specific to the intervention, that is, MT participants did a mindfulness exercise, and PST participants did a relaxation exercise or an imagery exercise. The two intervention groups did not differ in the amount of formal practice (*t* = 0.37, *p* = .71, d = .09; BF < 3). Participants of the WL were asked to complete posttest measures 4 weeks after the pretest. After this, these participants were randomly assigned to either the MT or PST intervention program.

The same person, who has a solid training in sports psychology and 7 years of experience in applied sport psychology, conducted all workshops. With all team sports (i.e., curling and floorball), the workshops were conducted separately for each team (i.e., each female curling team separately, each male floorball team separately, etc.). The sport psychologist went to the teams’ training facility, where the workshops took place in a meeting room. For the individual athletes (i.e., tennis and badminton), one workshop series (i.e., four workshops) was held per intervention condition (MT, PST, and WL). The workshops for the individual athletes took place in the performance center in which the sports psychologist was working. The group size in the workshops was three to ten participants (*M* = 6.0, *SD* = 2.11). Of the 64 participants in the MT and PST groups, 50 took part in all four workshop sessions. The remaining 14 participated in three of four workshops. Those who missed a workshop received the workshop materials (i.e., presentation slides, worksheets, homework, and audio files) by e-mail. The MT and PST groups did not differ in the number of workshops missed (*t* = .23, *p* = .82; d = .06; BF < 3).

### Measures

Table 2 gives an overview of the measures used in our study, including the Cronbach alphas of all scales for the pretest, Likert scale range, and example items. We used the short form of the Five Facet Mindfulness Questionnaire (FFMQ-SF, [9]) to assess mindfulness. Similar to other studies [3], we only administered the *acting with awareness*, *nonjudging of inner experience*, and *nonreactivity to inner experience* subscales of the FFMQ-SF because the other two subscales have been found to be less reliable (e.g., [13]). The use of psychological skills was assessed by the subscales *self-talk*, *imagery*, *goal setting*, *activation,* and *relaxation* of the Test of Performance Strategies (TOPS, [34]). For all TOPS scales, we calculated a mean score from the items that covered the training context and the competition context.

**Table 2:** Measures, Cronbach alpha values and example items

Concept	Measurement	Subscale(s)	α	Example item
Mindfulness	FFMQ-SF^a)^	*Acting with awareness*	.50	*I find it difficult to stay focused on what’s happening in the present moment (reversed).*
		*Nonjudging of inner experience*	.73	*I tell myself I shouldn’t be feeling the way I’m feeling (reversed).*
		*Nonreactivity to inner experience*	76	*I watch my feelings without getting carried away by them.*
Use of psychological skills	TOPS^a)^	*Self-talk*	.87	*I say things to myself to help my competitive performance.*
		*Imagery*	.79	*During practice, I visualize successful past performances.*
		*Goal setting*	.86	*I have very specific goals for training.*
		*Activation*	.81	*I can raise my energy level at competitions when necessary.*
		*Relaxation*	.87	*When the pressure is on at competitions, I know how to relax.*
		*Techniques to handle emotions*	.65	*In competition, I use techniques to control my emotions.*
		*Techniques to regulate attention*	.72	*In training, I use techniques to control my attention.*
Emotion regulation	TOPS^a)^	*Emotional control*	.83	*My emotions keep me from performing my best at competitions (reversed)*
Experiential avoidance	AAQ-II^b)^	–	.79	*I’m afraid of my feelings.*
Emotional competencies	SEC-27^a)^	*Acceptance of emotions*	.64	*I am able to stand by my feelings.*
Attention regulation	TOPS^a)^	*Attention control*	.83	*I am able to control distracting thoughts when training.*
Cognitive interference	TOQS^b)^	*Thoughts of escape*	.90	*During competition, I have thoughts that I want to get out of here.*
		*Situation-irrelevant thoughts*	.85	*During competition, I have thoughts about what I’m going to do later in the day.*
		*Performance worries*	.77	*During competition, I have thoughts that I’m having a bad day.*
	TOPS^a)^	*Negative cognitions*	.87	*My self-talk during competition is negative.*
Decentering	EQ^a)^	*Distanced perspective*	.77	*I can separate myself from my thoughts and feelings.*
Dealing with failures	ASOAF6^b)^	*Action orientation*	.63	*If I get little playtime to prove myself, and something goes wrong, I try to do better in the next action.*
				*If I fail in an important situation in a (championship) match, I quickly forget about it and concentrate on the next chance.*
				*If everything goes wrong in one day, then I continue to play as determinedly as if it hadn’t happened.*
		*State orientation*	.83	*If I fail in an important situation in a (championship) game, then it goes through my mind repeatedly in the further course of the game.*
				*If the coach criticizes my behavior, then it keeps me busy even during the game.*
				*If I fail several in actions one after the other in a (championship) game, then my thoughts circle around these failed actions for a long time.*

*Note*. *FFMQ-SF* Five Facets Mindfulness Questionnaire short form, *TOPS* Test of Performance Strategies, *AAQ-II* Acceptance and Action Questionnaire, *SEC-27* Self-assessment of Emotional Competencies, *TOQS* Thought Occurrence Questionnaire for Sport, *EQ* Experience Questionnaire, *ASOAF6* Action and State Orientation after Failure

^a)^ = 5-point Likert scale, ^b)^ = 7-point Likert scale

The TOPS subscales *emotional control* and *attentional control* involve two types of items: items that assess whether athletes use techniques to control emotions and attention, and items that assess whether athletes are successful in controlling their emotions and attention. We separated the two types of items, which resulted in four adapted scales: *use of techniques to control emotions*, *use of techniques to control attention, emotional control,* and *attention control* [8].

We used the adapted TOPS *emotional control* subscale to assess whether emotions interfere with athletic performance in training and competition We used the Acceptance and Action Questionnaire II (AAQ-II, [10]) to assess experiential avoidance and the acceptance subscale of the Self-Assessment of Emotional Competencies (SEC-27, [4]) to assess the ability to accept emotions.

We assessed *attention control* in training and competitions, using the adapted subscale of the TOPS. *Cognitive interference* was measured by the Thought Occurrence Questionnaire for Sport (TOQS, [32]) and the *negative cognitions* subscale of the TOPS [34]. The *distanced perspective* subscale of the Experience Questionnaire (EQ, [14, 16]) was used to assess decentering.

Based on the subscale for action orientation after failure by Beckmann [1], we developed two short scales to measure *action orientation after failure* and *state orientation after failure* (ASOAF6; see Table 2 for the items). Both scales were only slightly correlated (*r* = −.30) indicating that they were sufficiently independent. Furthermore, action orientation was highly correlated with optimism (*r* = .54; for state orientation *r* = −.45), whereas state orientation was highly correlated with choking (*r* = .44; for action orientation *r* = −.34). Thus, both scales were differentially associated with other performance-relevant factors (Horvath S: A A short scale to measure action-orientation and stateorientation after failure in soccer, in preparation).

### Statistical analysis

For the statistical inferences, a Bayesian approach was applied. The Bayesian method is well suited to deal with uncertainties of small samples and, briefly, works as follows: First, prior distributions for the quantities of interest (e.g., the intervention effect on a variable) are modeled. These priors represent our knowledge before looking at the data. Second, the prior distributions are updated by the observed data, yielding the posterior distributions – our knowledge of the effects after taking the data into account. Based on a posterior distribution, which, importantly, represents not only a point estimate of the effect but also its uncertainty, inferences about the effects are made: Posterior means (i.e., the point estimate of the effect) and 95% highest posterior density (HPD) intervals (i.e., the 95%-credence that the true effect lies within the interval) are estimated.

Our analysis focused on the observed pre-post-changes (i.e., differences from pre to post intervention) in all the variables. Markov Chain Monte Carlo sampling using the Python “PyMC3” implementation [33] of the Metropolis-Hastings algorithm was used to compute a) the posterior distributions of the pre-post-changes for all variables and interventions, respectively, and b) the posterior distribution for the difference of the pre-post-change in each variable between different interventions (e.g., the difference in pre-post-change of self-talk between PST and WL). The probability of PST and MT having an effect on a variable (i.e., the probabilities of the changes in PST and MT being different from those in WL and from each other) were determined via the fraction of the area under the posterior density curve where effect (x-value) > 0. The means of the effect-posteriors and the corresponding 95% HPD intervals were also derived from the posterior distributions of. The purpose of a HPD interval (or, more generally, a credible interval) is to summarize the knowledge about the parameters of interest. The 95% HPD interval represents the smallest value interval that spans 95% of the area under the posterior density curve. Its interpretation is thus straightforward: given the assumed prior and the observed data, the effect has 95% probability of falling within the 95% HPD interval. We thus chose to report the HPD intervals for the differences between interventions as they allow an intuitive assessment of the magnitude and the uncertainty of the effects. The likelihoods of the observed changes in the measured variables were modelled as normal distributions with uninformative, uniformly distributed priors for the means (limits of uniform distribution chosen based on Likert scale of respective variable) and a half-normally distributed prior (SD = 10) for the standard deviation. Uninformative priors were used in order to eliminate potential bias arising from false prior assumptions. For comparability between the different variables, the observed posterior means and HPD intervals were scaled to (i.e., divided by) the range of the Likert-scale in the respective variable.

## Results

**Fig. 2:**
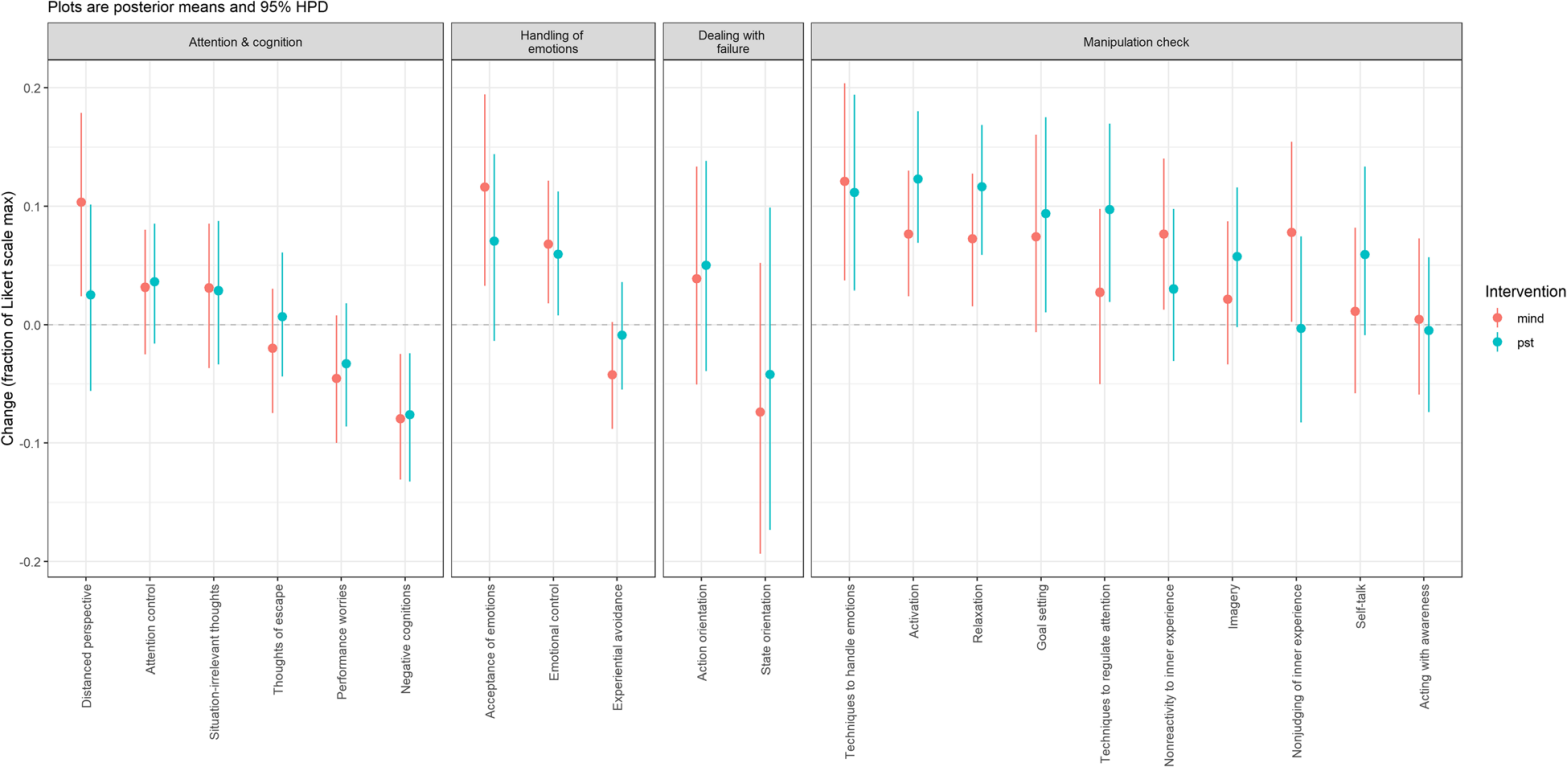
Observed effect-posterior means and HPD intervals (scaled to the range of the Likert-scale in the respective variable). Example: The mean of the effect-posterior of *distanced perspective* for MT is .10 and the 95% HPD interval ranges from 0.02 to 0.18. This means that compared to WL, in MT we observed an increase in *distanced perspective* of 10% on the corresponding Likert scale and with a credence of 95% the true effect lies within 0.02 and 0.18

Due to the large scope of the results, we will include a separate short discussion for each of the four parts (i.e., manipulation check, handling of emotions, attention and cognition, and dealing with failure). In the general discussion, the results will then be merged into an overall picture. An overview of all results is provided in Table 3 and Fig. 2. Table 3 shows the probabilities of PST and MT having effects on the analyzed variables (i.e., the changes of the variables in the PST and MT being different from the WL and from each other). Figure 2 includes the means of the effect-posteriors and the corresponding 95% HPD intervals for MT and PST compared to WL (scaled to the range of the Likert-scale in the respective variable).

**Table 3:** Probabilities of PST and MT having effects on the analyzed variables

Concept	Measurement	Subscale(s)	Probabilities
Mindfulness	FFMQ-SF	*Acting with awareness*	MT > WL = 57%; PST > WL = 41%; MT > PST = 66%
		*Nonjudging of inner experience*	**MT > WL = 98%**; PST > WL = 43%; **MT > PST = 98%**
		*Nonreactivity to inner experience*	**MT > WL = 98%**; PST > WL = 81%; MT > PST = 92%
Use of psychological skills	TOPS	*Self-talk*	MT > WL = 58%; **PST > WL = 95%**; PST > MT = 91%
		*Imagery*	MT > WL = 76%; **PST > WL = 98%**; PST > MT = 87%
		*Goal setting*	**MT > WL = 95%**; **PST > WL = 99%**; PST > MT = 68%
		*Activation*	**MT > WL = 100%**; **PST > WL = 100%**; **PST > MT = 96%**
		*Relaxation*	**MT > WL = 100%**; **PST > WL = 100%**; **PST > MT = 96%**
		*Techniques to handle emotions*	**MT > WL = 100%**; **PST > WL = 100%**; PST > MT = 38%
		*Techniques to regulate attention*	MT > WL = 75%; **PST > WL = 100%**; **PST > MT = 96%**
Emotion regulation	TOPS	*Emotional control*	**MT > WL = 99%**; **PST > WL = 99%**; MT > PST = 63%
Experiential avoidance^a^	AAQ-II	–	**MT > WL = 96%**; PST > WL = 65%; MT > PST = 92%
Emotional competencies	SEC-27	*Acceptance of emotions*	**MT > WL = 100%**; **PST > WL = 96%**; MT > PST = 88%
Attentional regulation	TOPS	*Attention control*	MT > WL = 89%; PST > WL = 91%; MT > PST = 46%
Cognitive interference^a^	TOQS	*Thoughts of escape*	MT > WL = 78%; PST > WL = 38%; MT > PST = 84%
		*Situation-irrelevant thoughts*	MT > WL = 18%; PST > WL = 17%; MT > PST = 49%
		*Performance worries*	**MT > WL = 95%**; PST > WL = 88%; MT > PST = 71%
	TOPS	*Negative cognitions*	**MT > WL = 100%**; **PST > WL = 100%**; MT > PST = 58%
Decentering	EQ	*Distanced perspective*	**MT > WL = 100%**; PST > WL = 74%; **MT > PST > 98%**
Dealing with failures	ASOAF6	*Action orientation*	MT > WL = 82%; PST > WL = 89%; MT > PST = 41%
		*State orientation*^a^	MT > WL = 84%; PST > WL = 71%; MT > PST = 64%

Note. *MT* Mindfulness intervention, *PST* Psychological skills training intervention, *WL* Wait-list control group, *FFMQ-SF* Five Facets Mindfulness Questionnaire short form, *TOPS* Test of Performance Strategies, *AAQ-II* Acceptance and Action Questionnaire, *SEC-27* Self-assessment of Emotional Competencies, *TOQS* Thought Occurrence Questionnaire for Sport, *EQ* Experience Questionnaire, *ASOAF6* Action and State Orientation after Failure; probabilities of 95% or higher are highlighted in bold, ^a^ = we expected these concepts to decrease trough MT and/or PST, the probability indicates accordingly whether there has been a *reduction* in comparison between the groups

### Results and discussion: manipulation check

Two aspects of mindfulness, i.e., *nonjudging of inner experience* and *nonreactivity to inner experience*, improved in MT compared to both WL (probabilities > 98%) and PST (probabilities > 92%). The finding that MT was also better than PST in these measures implies that the changes were due to the specific content of the mindfulness workshops and cannot be explained simply by the fact that any intervention has taken place. The probability that the *acting with awareness* aspect of mindfulness improved in MT compared with WL was only 57 and 66% compared with PST. Thus, in our workshop setting it seemed easier to teach the other two aspects of mindfulness. In order to change *acting with awareness*, it would probably have required more formal mindfulness training. In consequence, MT passed the manipulation check with the exception of one scale.

Compared with the WL, PST improved the use of all techniques and in the use of techniques to control emotions and attention compared with the WL (probabilities > 95%). PST was also superior to MT (probabilities > 91%) with the exception of *goal setting, imagery* and the *use of techniques to control emotions*. The superiority of PST over MT was most evident in arousal regulation (*activation* and *relaxation*) and in the *use of techniques to control attention* (probabilities > 96%). This last result may be related to the fact that techniques to control attention were trained much more explicitly in the PST program (e.g., by using self-talk to remind oneself of process goals to increase concentration), whereas MT mainly aimed at generally increasing attention. Overall, the PST clearly passed the manipulation check.

Regarding the use of some techniques (i.e., *goal setting*, *activation*, *relaxation*, and *use of techniques to control emotions*) PST *and* MT were better than WL (probabilities > 95%). We did not expect this shared effect. For MT, this shared effect can be considered a “side effect”, supported by the finding that PST was still better than MT. In *goal setting*, a possible explanation is that MT led the athletes to approach their training and competition with the goal to stay in the present, and in *activation* and *relaxation*, an explanation could be the improved perception of inner states through mindfulness and a corresponding adaptation of the state to current needs. Mindfulness is, in itself, a technique to handle emotions, which may explain corresponding changes in MT when it comes to the use of techniques to deal with emotions. However, we did not see the reverse effect as clearly, namely, that PST improved mindfulness: the probability that PST was better than WL in *nonjudging of inner experience*, and *acting with awareness* was lower than 44%, but the probability that PST was better than WL in *nonreactivity to inner experience* was 81%. In sum, there appeared to be differential intervention effects: whereas PST improved psychological skills, MT improved mindfulness. However, we also saw a broad treatment effect especially for MT, which was indicated by the use of individual psychological techniques that were also improved in the MT.

The mean effect of PST on all psychological skills and use of techniques was .09 points on the Likert scale (scaled to the range of the Likert-scale, i.e., an increase of 9%). The mean effect of MT on all three aspects of mindfulness was .05. The lower number is due to the acting with awareness scale that did not show any changes.

### Results and discussion: handling of emotions

Compared with WL, both MT and PST improved *emotional control* in training and competition (probabilities > 99%), but did not differ from each other (probability that MT was better than PST = 66%). The observed effect-posterior means were .07 for MT and .06 for PST. The *emotional control* scale of the TOPS essentially measures whether emotions interfere with athletic performance in training and competition. This can be interpreted as an example of the shared effect of different forms of mental training. Our results seem to show that athletes can reach the same goal (non-interference of emotions) using different strategies, i.e., mindfulness or the use of psychological strategies.

Compared with WL and PST, MT showed reduced *experiential avoidance* (probabilities > 92%). This made sense since mindfulness interventions aim to help people to feel all experiences, including the unpleasant ones, without avoiding them. In PST, experiential avoidance was not addressed in the workshops, which explains the finding that the probability of a decreased experiential avoidance in PST compared with WL was only 65% and an observed effect-posterior mean of − 0.01.

MT was also clearly superior to WL when it came to the ability to *accept emotions* (probability > 99%), the probability that MT was superior to PST was not as big (88%). The results show that PST also improved the ability to *accept emotions* compared with the WL (probability = 96%). The improvement in acceptance in PST was probably another “side effect”. This is underlined by the observed effect-posterior means (.12 for MT and .07 for PST). A possible explanation for these changes, although acceptance was not addressed during the PST intervention, could be group effects. For example, when participants in the workshops saw that other athletes had similar emotions to their own, they may have been able to see their own feelings as normal and have been more likely to accept them.

### Results and discussion: attention and cognition

As expected, the two interventions did not differ regarding improvements in attention control in training and competition (probability that MT was better than PST = 46%), and the probability that the two interventions improved compared with WL was comparable: 89% for MT and 91% for PST. Therefore, compared with WL both intervention groups showed improvements in attention control with about 90% probability. This is another example of the shared effect of different forms of mental training, and apparently, there are several ways to improve attention (PST and MT).

The results regarding cognitive interference were mixed. The probability that the interventions compared to the WL reduced interference by *situationally irrelevant thoughts* was only 18% or lower. The probability that MT reduced interference by *thoughts of escape* was 78% compared with WL, and 84% compared with PST. Results were clearer for interference by *performance worries* and *negative cognitions*. Here, the probability that MT improved these was higher than 95% compared with the WL and 71% higher compared with PST. The probability that PST improved *performance worries* and *negative cognitions* compared with WL was 88% and almost 100% respectively. Both MT and PST therefore appeared to have a shared effect on certain aspects of cognitive interference (i.e., worries and negative cognitions).

As expected, we also saw a clear pattern for the increase of decentering in favor of MT, i.e., MT was better than WL and PST (probabilities > 98%). MT seemed to help athletes to distance themselves more easily from internal processes such as thoughts or feelings. This is an important prerequisite for psychological flexibility, that is, the ability to pursue one’s goals even when unpleasant thoughts and feelings are present [20]. Within attention and cognition, we see the greatest observed effect-posterior means for *negative cognitions* (− .08 for MT and PST) and distanced perspective (.10 for MT).

### Results and discussion: dealing with failures

The results for *action orientation*^2^ show that the two interventions improved this with a probability of 82% (MT) and 89% (PST) compared with WL. The comparison of the two interventions indicated that the probability that PST was better than MT was only 59%. *State orientation* decreased in the MT compared with the WL with a probability of 84%, and the probability of a decrease in the PST compared with the WL was 71%. The comparison of the two interventions showed that the probability that MT was better than PST was only 64%. The observed effect-posterior means for *action orientation* were .04 in MT and .05 in PST; *for state orientation* they were − .07 in MT and − .04 in PST. The HPD interval was the largest for *action orientation* and *state orientation*, indicating more uncertainty about where the true effect lies. Thus, both interventions had a similar probability for effects on action and state orientation. This is yet another example of a shared effect of different forms of mental training.

## Discussion

In this study, we investigated the effect of two forms of mental training on different psychological parameters relevant to athletic performance. It is the first RCT to include a mindfulness training intervention (MT), a psychological skills training intervention (PST) and a waitlist control group (WL). Both interventions passed the manipulation check, that is, MT led to an increase in mindfulness, and PST led to an increased use of psychological strategies in the respective sports. Both interventions had the expected positive effects on performance-related factors, such as the handling of emotions and the control of attention. We found both differential and shared effects. By differential effects, we mean that only one of the two mental training forms was effective (i.e., led to changes) for a given outcome variable. By shared effects, we mean that both interventions were effective for a certain outcome variable. In some cases, we expected these shared effects, and in others, they occurred more in the form of “side effects.” We have structured the discussion so that we first discuss shared effects that we expected, then shared effects that we did not expect and then differential effects.

As expected, we found shared effects in dealing with emotions, in attention control, and in dealing with mistakes. All concepts were assessed using self-report questionnaires. In detail, we found that both interventions led to an improved handling of emotions, which was indicated by our results that emotions were less likely to interfere with performance in training and competition. Both, MT and PST also led to an increase in attention control. This indicates that after these interventions athletes in training and competition were less distracted by internal or external events and better able to maintain their focus. The effect of improvement in attention control was somewhat lower in both interventions compared with the improved handling of emotions. Thus, it appeared that our interventions had a greater impact on the handling of emotions than on attention control. We also saw a tendency for improvements by the interventions regarding action vs. state orientation (the latter considered being less helpful), although the results were less clear than for the other variables. This may be related to the constructs of action vs. state orientation being less closely related to the interventions compared with the other outcome variables and were possibly influenced by other factors than mental training.

For some variables, we found shared effects that we did not expect. We called them “side effects,” when both intervention groups showed effects, but the intervention group for which we expected the effect was still superior to the other. Such “side effects” were the increased use of individual mental strategies (i.e., goal setting and arousal regulation) shown by the MT group, and the increased acceptance of emotions and, to a lesser extent, to not react automatically to inner experiences shown by the PST group. These results suggest that sport psychological interventions often seem to have effects that are not directly intended. Future research should investigate these effects in more detail. Psychotherapy research could serve as a model here. For some time, psychotherapy research has been investigating which factors, apart from the psychotherapeutic approach itself, influence the outcome of the therapy [27]. Common examples of such common factors are the therapeutic alliance, affective experiencing or patient engagement [37].

We found differential effects for some variables. For these variables, only one of the two interventions was effective. This was evident in the use of self-talk, where we only found effects for the PST. In contrast, we found differential effects in favor of MT in the ability to look at internal processes from a certain distance, without judging and without avoiding unpleasant processes. This clarity and acceptance of inner experiences seems to be fostered especially through MT. If these two processes prove to be particularly performance relevant in sport, sport psychologists are well advised to promote them in their interventions. However, further research is needed to highlight this possible impact. The differential effects we observed in this study were not surprising in that they were very close to the intervention itself. Future research could investigate whether there are other outcome variables on which various forms of mental training have differential effects. One such variable would be well-being. Mindfulness interventions seem to be more likely to have effects on well-being and other mental health parameters than psychological skills training interventions [18].

The present study has certain limitations, the most important of which is study planning and implementation. As shown in the supplementary material, we were not able to implement everything as planned. It is particularly significant that we did not record specific indicators for physical performance. This means that we cannot determine whether the psychological variables being recorded and declared as relevant to performance really promote athletic performance. Future research should therefore not only include psychological variables but also indicators of athletic performance (objective measures as well as external and self-assessment of performance). In addition, our results solely rely on self-report data, which increases the risk of method biases [29]. While our study investigated which mental training works for which outcome, future research should additionally consider potential moderators. This would mean to also investigate which mental training works for whom and under what circumstances. Future research should also examine how meaningful changes such as the ones we observed are, either by evaluating the subjective athletes’ perspective or by comparing changes in psychological variables to changes in objective performance data. A disadvantage of conducting the workshop in the way we did was that our method proved to be very time-consuming and was limited regarding time and place. It would be instructive to examine alternative forms of interventions that do not have these disadvantages, for example, forms of online interventions [35].

## Conclusion

We conclude that both forms of mental training lead to expected changes in performance-relevant psychological factors. The results of our study suggest that different paths may lead to the desired outcomes, and accordingly, both forms of mental training seem justified. Both interventions enhanced attention control, handling of emotions, and the functional behavior after failure. MT seems to have advantages over PST when it comes to a distanced perspective to once inner experience (i.e., defusion) and to less avoidance and more exposure of emotions. Sport psychologists may not ask themselves whether they should do either mindfulness or psychological skills training. Instead, they should consider the demands of the respective sport as well as which outcome they want to target and choose the most appropriate interventions based on empirical evidence, their personal training and their and the athletes preferences.

## Supplementary information

**Additional file 1.**

## Data Availability

The data is available from the corresponding author on reasonable request.

## Abbreviations

AAQ-II: Acceptance and Action Questionnaire
BSI: Brief Symptom Inventory
DSS: Decentering Scale for Sport
EQ: Experience Questionnaire
FFMQ-SF: Five Facets Mindfulness Questionnaire Short Form
MT: Mindfulness training intervention
PST: Psychological skills training intervention
RCT: Randomized controlled trial
SEC-27: Self-Assessment of Emotional Competencies
TOPS: Test of Performance Strategies
TOQS: Thought Occurrence Questionnaire for Sport
WL: Wait-list control group

## Footnotes

1 The study slightly deviates from the study protocol in a few ways. We have submitted an overview of the changes in the supplementary material section.

2 Only team sport players filled out the action and state orientation survey.
