# Supplementary material for: Differential and shared effects of psychological skills training and mindfulness training on performance-relevant psychological factors in sport: a randomized controlled trial
Source: BMC Psychol. 2020 Aug 6;8:80. doi: 10.1186/s40359-020-00449-7 (PMC7409666; doi:10.1186/s40359-020-00449-7)
Supplement: Supplementary file 1 — Additional file 1. [file 40359_2020_449_MOESM1_ESM.docx]

**Supplementary material**

The present study differs to some degree from the description in the study protocol (Röthlin et al., 2016). On the one hand, we had to adapt some minor points for the method (sample, procedure, and measurements). On the other hand, we decided to drop the Functional Athletic Behavior (FAB) measure from the article because it did not work as intended. In this supplementary material section, we address both issues.

**Deviations from study protocol**

**Method section**

As planned, our sample covered curling, tennis, and floorball, but we did not include volleyball. This was necessary to reduce the administrative effort because, in Switzerland, we cannot cover the sports through students/colleges, but have to go through the individual federations each time.

When we started preparing the study material, we decided to reduce the athletes’ participating time – to increase their compliance motivation – by reducing the number of instruments. We dropped the measurements that are time-consuming (e.g., the attention network test) and were less relevant and/or already covered by other instruments (e.g., athletic mindfulness questionnaire). Even with the shortened version, we had to remind participants repeatedly to complete the post-intervention survey. Consequently, we decided to withdraw the follow-up measurement.

**Results section**

We decided to focus on the main analyses, namely manipulation (e.g., changes in mindfulness or use of strategies such as self-talk) and main outcome variables. We wanted to simplify the study and to refocus on the main questions and comparisons, namely, the effectiveness of both psychological skills training and mindfulness-based interventions in competitive sports.

**Problems with the FAB instrument**

As described in the study protocol (Röthlin et al., 2016), we intended to use a short questionnaire to measure FAB during a game or a competition (ambulatory assessment). Although we put a lot of effort into the development and piloting of the FAB questionnaire, we doubted its appropriate use in this study (see below). To measure FAB, we used the three items we already mentioned in the study protocol:

1. Rate regardless of the result or outcome: In the last sequence, my movements and actions were of a high quality (precise, energetic, well timed, etc.).

2. Rate regardless of the result or outcome: In the last sequence, I was focused on the task.

3. Rate regardless of the result or outcome: In the last sequence, I behaved on the pitch/field/ice as the athlete that I would like to be.

To assess their FAB, athletes rated each question from 0 (no agreement) to 100 (total agreement) by placing a cross on a rating line. Unfortunately, we could not use mobile phones for this ambulatory assessment, as the use of mobile phones is not allowed in most sports during competitions. Thus, we had to use a paper-pencil version instead. Depending on the sport, athletes were asked to complete the FAB three to four times during the competition.

We pilot tested the paper-pencil version in different sports. The FAB was introduced verbally to guarantee that participants understood the items and their task. As intended, all athletes understood the instructions and questions, and very few of the athletes felt disturbed by answering them during the competition.

When using the FAB in our study, we kept the verbal instructions and added a familiarization run (competition or training game). Although there were no problems in the beginning, there were some small changes in the implementation later in the course. Some athletes completed some measurements after the competition (e.g., if they did not have enough time), or they made temporary notes by writing on the back of their hands. However, in sum, the FAB is a usable instrument to capture performance-relevant factors close to the competition.

In the present study, the FAB was intended to be used to examine the effect of the interventions (PST and MT) on performance, independent of the result of the competition (victory or defeat). Based on this assumption, we decided to measure FAB repeatedly during the same competition. In retrospect, it appears that it would have been better to record several competitions in order to answer the question whether our interventions (PST and MT) had an effect on FAB.

**Conclusion**

Thus, we believe that the FAB is an important approach to the measurement of physical performance, allowing performance to be measured independent of the outcome. Unfortunately, the way we used the FAB in our study (single competition) did not allow us to evaluate the effect of the two interventions (PST and MT) relative to the WL group.
